# Factors Influencing *Salmonella enterica* Contamination and Multidrug Resistance in Pork Available at Modern Retail Stores in Urban Southern Thailand

**DOI:** 10.3390/biology15110853

**Published:** 2026-05-29

**Authors:** Teerarat Prasertsee, Witaya Suriyasathaporn

**Affiliations:** 1 Faculty of Veterinary Science, Prince of Songkla University, Songkhla 90110, Thailand; teerarat.p@psu.ac.th; 2 Veterinary Academic Office, Faculty of Veterinary Medicine, Chiang Mai University, Chiang Mai 50100, Thailand; 3 Research Center of Producing and Development of Products and Innovations for Animal Health and Production, Chiang Mai University, Chiang Mai 50100, Thailand; 4 Oversea Campus, Asian Satellite Campuses Institute, Nagoya University, Nagoya 464-8601, Japan

**Keywords:** antimicrobial resistance, modern retail store, pork, *Salmonella* spp., urban area

## Abstract

Background: *Salmonella* is a foodborne pathogen that causes human gastroenteritis and is transmitted through the livestock production chain, with contamination occurring in pork across modern retail stores. In this study, we aimed to determine the prevalence of *Salmonella* contamination in pork, characterize antimicrobial resistance (AMR) profiles, and identify associated risk factors in urban southern Thailand. Methods: A total of 600 pork products were collected and tested for *Salmonella* identification by using ISO 6579:2017 and antimicrobial susceptibility. Associations between contamination and risk factors were assessed by Fisher’s exact test. A final model was developed using repeated logistic regression with a Generalized Estimating Equation (GEE) approach. Results: The prevalence of *Salmonella* contamination was 33.33%, with the serotype Rissen most frequently detected. Store type, packaging type, marketing target, and seasonal variation were significantly associated with contamination. Sealed packaging and products intended for household use were associated with lower contamination risk. High resistance was observed for ampicillin and tetracycline, while the rate of multidrug resistance was 12.5%, and 10.5% of the isolates were pan-susceptible. Conclusions: *Salmonella* contamination varies across pork products and retail conditions; proper handling and thorough cooking are essential. From a One Health perspective, these findings highlight links between animal production, food systems, and human health, supporting integrated strategies to reduce transmission and antimicrobial resistance.

## Simple Summary

*Salmonella* is a foodborne bacterium that can be carried by livestock animals such as pigs and transmitted to humans through contaminated pork, representing an important public health concern within the One Health framework, which recognizes the interconnected health of humans, animals, and the environment. This study aimed to estimate the prevalence of *Salmonella* contamination in pork sold in modern retail stores in urban southern Thailand and to identify associated risk factors, while also assessing antimicrobial resistance profiles of the isolates. A total of 600 pork samples were analyzed, and approximately one-third were positive for *Salmonella*. Contamination was significantly associated with packaging type, product marketing purpose, and seasonal variation, indicating that both supply chain practices and environmental conditions influence food safety outcomes. Antimicrobial susceptibility testing showed common resistance to ampicillin and tetracycline, while multidrug resistance remained relatively low. These findings highlight that *Salmonella* contamination in pork is influenced by multiple linked factors across animal production, retail handling, and consumer products, reinforcing the need for integrated One Health approaches. Strengthening food safety practices along the pork production and distribution chain is essential to reduce foodborne infection risks and limit the spread of antimicrobial resistance to humans.

## 1. Introduction

Nontyphoidal *Salmonella* (NTS) is a major foodborne pathogen causing gastroenteritis in both humans and animals. It can result in a range of clinical symptoms, including septicemia, enterocolitis, and even subclinical infections, all of which depend on the host’s immune status. Although NTS typically does not lead to severe illness in healthy individuals, it is important to provide antimicrobial treatment for severe cases, especially among high-risk groups such as children under five, immunocompromised patients, and the elderly [1,2]. In major urban centers like Bangkok, Ho Chi Minh City, Jakarta, and other major urban cities, the vast food supply chain heightens the risk of *Salmonella* and antimicrobial resistance (AMR). While Bangkok remains unparalleled, the smaller major urban centers, Songkhla and its economic hub, Hat-Yai, consistently rank as the primary urban centers of Southern Thailand and are frequently identified as the fourth- or fifth-largest cities in the nation based on urban population metrics [3]. As in primary urban cities worldwide, most people secure their health by purchasing food products from modern retail stores, where the products are produced in factories certified to national standards.

*Salmonella* in pork serves as a crucial indicator within the One Health paradigm, highlighting the interconnectedness of veterinary medicine, environmental science, and public health. In swine production, pigs frequently act as asymptomatic carriers of *Salmonella enterica*, shedding the bacteria into their environment through feces. The environmental persistence of this pathogen enables it to re-enter the food chain, posing a significant zoonotic risk during the slaughter and processing of pork products [4,5]. Central to the further spread of *Salmonella* is the crisis of AMR; the use of antibiotics in livestock can select for multidrug-resistant strains that are subsequently transmitted to humans through meat consumption, complicating medical treatments and increasing mortality [6,7]. A One Health approach, therefore, shifts the focus from merely treating human illness to integrated surveillance across all sectors, implementing on-farm biosecurity, improving slaughter hygiene, and monitoring environmental reservoirs to safeguard global health [6,8]. In urban settings, the One Health relationship between pork and *Salmonella* becomes increasingly intricate, influenced by high population density, extended food supply chains, and the prevalence of “wet market” culture (traditional open-air marketplace).

The journey from slaughter to the dinner plate in a bustling downtown involves numerous points at which food safety can be compromised. For example, maintaining a constant temperature of 4 °C is difficult in tropical urban environments, leading to a significant increase in *Salmonella* cross-contamination from the slaughterhouse to reach levels that pose a dangerous infectious dose [9]. In Chiang Mai, Thailand, prevalence in supermarkets was lower (9.8%) than in wet markets (73.2%), highlighting that the effectiveness of “modern” retail safety varies by region and management [10]. In contrast, in a study of urban Hanoi, supermarkets had a *Salmonella* prevalence of 82.9%, higher than that in traditional wet markets [11]. The higher rates in modern outlets compared to traditional markets might be due to extended storage times or centralized processing. A rising concern in urban retail is the presence of antibiotic-resistant *Salmonella* strains, specifically those resistant to ampicillin (91.29%), tetracycline (88.26%), and streptomycin (84.47%). Over 80% of isolates in some urban retail chains are classified as multidrug-resistant, meaning they are resistant to three or more antimicrobial classes [12]. Various studies in Vietnam reported a high prevalence of microbial contamination across traditional pork value chains, from slaughterhouse (25% to 60% of pork samples were positive for *Salmonella*) [13,14] to different types of retail outlets (58% to 73% pork samples were positive for *Salmonella* [11,13,15,16].

The inconsistency in the severity of the *Salmonella* and AMR epidemic in modern retail stores in urban areas might also be caused by product characteristics, such as factory or store packaging, household or restaurant use, and grinding; or the types of modern retail stores. Therefore, the aim of this study was to determine the prevalence of *Salmonella* contamination in pork, characterize antimicrobial resistance (AMR) profiles, and identify the factors associated with *Salmonella* contamination in different marketed pork products originating from nationally certified factories and available in modern retail stores in the Songkhla municipality and Hat-Yai, Thailand.

## 2. Materials and Methods

### 2.1. Pork Sample Collection

**Figure 1:**
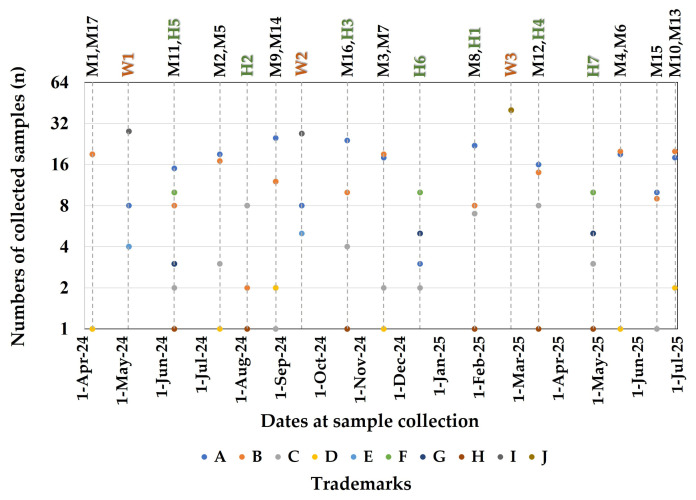
Sample collection schedule. Stores included minimarts (M1–M17), hypermarkets (H1–H7), and wholesale stores (W1–W3). Minimarts and hypermarkets were randomly sampled at 20 per store.

This cross-sectional study was conducted from April 2024 to July 2025 across 27 modern retail stores, including minimarts (*n* = 17), hypermarkets or supercenters (*n* = 7), and wholesale stores (*n* = 3), in the urban area of Songkhla province, southern Thailand. For each store, 20 samples (5 per type) and 40 samples (10 per type) were randomly collected from different trademarks within the same store for minimarts and hypermarkets, respectively, and for wholesale stores, 40 samples (10 per type) were collected equally from approximately one to two stores/month. The collection schedules are summarized in Figure 1. The types of samples included (1) factory-packaged unground pork, (2) factory-packaged ground pork, (3) unpackaged unground pork, and (4) unpackaged ground pork. The entire portion of factory-packaged pork was purchased, while the unpacked pork was scooped out in approximately 1 kg portions from the containers used to display pork cuts for sale in stores. Data on collection dates and sample details, including trademarks, product types, marketing targets (household use or restaurant use), and store areas, were recorded. Each sample was individually double-bagged before being placed in insulated boxes containing ice and transported to the laboratory within 4 h.

### 2.2. Bacterial Culture and Salmonella Identification

*Salmonella* was isolated in accordance with ISO 6579-1:2017(E) [17]: Microbiology of the food chain—Horizontal method for the detection, enumeration and serotyping of *Salmonella*, as a standard protocol. Twenty-five grams of the sample were placed in sterile bags using an aseptic technique. Each sample was mixed with 225 mL of buffered peptone water (BPW; Oxoid, Altrincham, UK), homogenized in a stomacher for 2 min, and incubated for 18 ± 2 h at 37 °C ± 1 °C. Three 33-μL drops of the incubated BPW were inoculated into modified semisolid Rappaport Vassiliadis medium (MSRV) with novobiocin supplement (Oxoid, Altrincham, UK) and incubated for 24 ± 3 h at 41.5 °C ± 1 °C (negative samples were re-incubated for an additional 24 ± 3 h). Presumptive *Salmonella*, identified by white/gray turbid zones radiating from the point of inoculation on MSRV, was streaked onto xylose lysine deoxycholate (XLD) medium (Oxoid, Altrincham, UK) and brilliant-green phenol-red lactose sucrose (BPLS) medium (Oxoid, Altrincham, UK). These were incubated for 24 ± 3 h at 37 °C ± 1 °C. Five suspected colonies, appearing red with a black center on XLD medium and red surrounded by a bright red zone on BPLS medium, were subcultured onto Nutrient agar (NA; Oxoid, Altrincham, UK) and incubated at 37 °C for 24 ± 3 h. The pure cultures on NA were subjected to biochemical testing for *Salmonella* spp. confirmation. Serological confirmation and serotyping were conducted on confirmed isolates according to the Kauffmann–White scheme.

### 2.3. Antimicrobial Susceptibility Testing and Minimum Inhibitory Concentrations

Susceptibility of all *Salmonella* isolates to different antimicrobial agents was assessed using Kirby–Bauer’s disk diffusion method. The *Salmonella* spp. pure cultures were picked and suspended in sterile saline and the turbidity was adjusted to the 0.5 McFarland standard. The suspension was streaked onto Mueller–Hinton agar (MHA; Oxoid, Altrincham, UK) using a sterile wet cotton swab in three directions; the plate was rotated approximately 60 degrees each time. Ten antimicrobial disks were placed (Oxoid, Altrincham, UK) on MHA: ampicillin AMP (10 μg), amoxicillin/clavulanate (AMC) (20/10 μg), chloramphenicol (CHL) (30 μg), ciprofloxacin (CIP) (5 μg), nalidixic acid (NAL) (30 μg), norfloxacin (NOR) (10 μg), streptomycin (STR) (10 μg), sulfisoxazole (SX) (250 μg), tetracycline (TET) (30 μg), and trimethoprim/sulfamethoxazole (SXT) (1.25/23.75 μg). Inhibition zones were measured according to the Clinical and Laboratory Standards Institute (2018) guidelines [18]. *Escherichia coli* ATCC 25922 was used as an internal quality control.

Resistance to colistin (COL) was determined using the gradient diffusion method according to the European Committee on Antimicrobial Susceptibility Testing guidelines (2021) [19]. COL resistance was interpreted using the EUCAST clinical breakpoints and epidemiological cut-off values in Annex 1 for *Salmonella* spp. (minimum inhibitory concentrations of ≤2 and >2 mg/L were considered sensitive and resistant, respectively).

### 2.4. Statistical Analysis

Data were presented as counts and percentages by trademarks of pork samples. Factors influencing the *Salmonella* contaminations in this study included grinding (ground, unground), factory packaging (packed, unpacked), types of store (minimarts, hypermarkets, or wholesale stores), season at collection based on the Songkhla province’s official website (mid-March to mid-May for summer and the rest for rainy), marketing targets (household or restaurant uses), and store areas (downtown or urban). Samples with *Salmonella* contamination were designated as contaminated and used as the dependent variable. The association between *Salmonella* contamination and its influencing factors was analyzed using Fisher’s exact test. The final model for factors associated with *Salmonella* contamination in pork was developed using backward repeated logistic regression with a Generalized Estimating Equations approach (Proc Genmod, SAS OnDemand for Academic (ver. 9.4)). The pork samples were the repeated subject variables, nested within the same stores, which were defined as the clustering unit. The correlation structure was exchangeable. The factor with *p* < 0.05 was allowed to enter, and remained in the final model with *p* < 0.05. Descriptive statistics were used to analyze antibiotic resistance. Antibiotics were classified into six groups: beta-lactams (AMP, AMC), chloramphenicol (CHL), quinolones (CIP, NOR, NAL), aminoglycosides (STR), sulfonamides (SX, SXT), and tetracyclines (TET). Multidrug resistance (MDR) was defined as resistance to at least three antibiotic classes in the same isolates.

## 3. Results

The number of samples of each type according to their trademark is demonstrated in Table 1. In total, ten pork trademarks were included in the study, and all products met the GMP standard. The A trademark was the most collected sample and was available in all stores and across all product types. The trademarks E, I, and J were intended for the restaurant’s use and were available through the wholesale stores. The trademarks D, E, G, and H were available only for factory-packaged pork, whereas F was available only for unpackaged products. 

### 3.1. Percentages of Salmonella Contamination and Serotype Distribution

**Figure 2:**
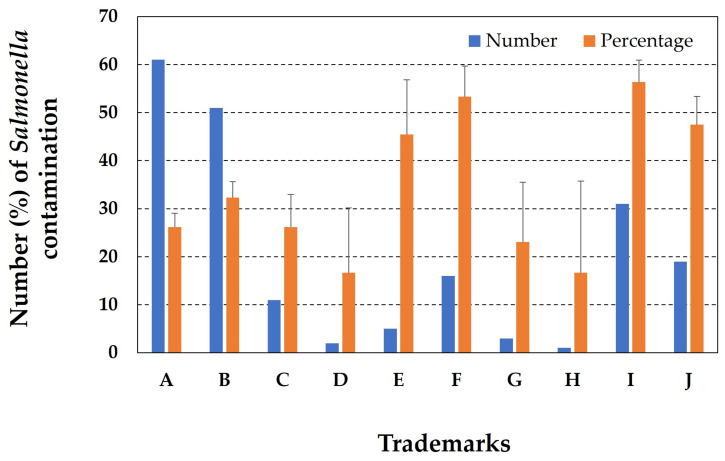
*Salmonella* contamination in pork products, presented as numbers and percentages with standard error of means (SE), in Songkhla province based on trademarks.

Of the 600 samples, 200 (33.33%) were contaminated with *Salmonella*. The numbers and percentages of *Salmonella* contamination among pork trademarks are shown in Figure 2. All trademarks were contaminated with *Salmonella*, from one sample in H (16.67%) to 61 samples (26.18%) in A. The highest *Salmonella* contamination rates were 45.45%, 47.5%, 53.33%, and 56.36% for Trademarks E, J, F, and I, respectively. Trademarks E, I, and J were available in wholesale stores and in the restaurant market, whereas F was available only in unpackaged pork.

Fourteen serotypes were identified in the *Salmonella*-contaminated samples. The serotype distribution of *Salmonella* enterica (*n* = 200) is presented in Table 2. *S. Rissen* (39%) was the serotype most frequently found in this study, followed by *S. Typhimurium* (19.5%) and *S. Enteritidis* (12.5%), respectively. *S. Rissen* was found in samples of almost all trademarks, with the exception of trademark D. Trademarks G and H were the only products that were free from both *S. Enteritidis* and *S. Typhimurium*.

**Figure 3:**
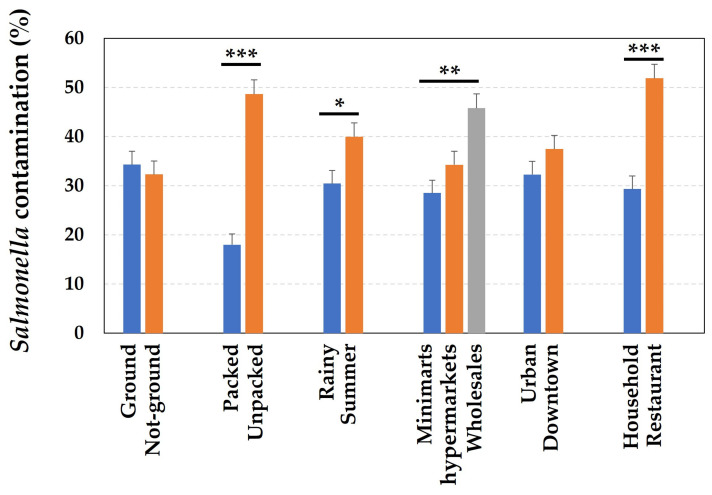
Percentages and standard error of means (SE) of the factors associated with *Salmonella* contamination in pork products in Songkhla province, Thailand. *, **, *** indicates significant associations at *p* < 0.05, <0.01, and <0.001, respectively.

Figure 3 illustrates the prevalence of *Salmonella* contamination across various factors, including grinding, factory packaging, store types, collection season, marketing targets, and store area. Statistical analysis indicated that grinding (ground and unground) and store area (downtown and urban) were not associated with *Salmonella* contamination. Pork with factory packaging (18.0%) had lower *Salmonella* contamination than unpackaged pork (48.67%) at *p* < 0.001. Household products (29.35%) had a lower contamination rate than pork for restaurants (51.89%), and *Salmonella* contamination was associated with store types (*p* < 0.01); pork from wholesale stores had the highest contamination rate (45.83%). Samples collected in summer had a higher contamination rate than those collected in the rainy season (*p* < 0.05). The final model of the factors influencing *Salmonella* contamination is shown in Table 3. The unpackaged pork had approximately four times the risk of *Salmonella* contamination compared with the factory-packaged products (OR = 4.306). Products for household use had a lower risk of contamination than those for restaurant use (OR = 2.540).

### 3.2. Antimicrobial Resistance of Salmonella Isolates

Percentages of antibiotic resistance in *Salmonella*-contaminated pork products collected from Songkhla province, Thailand, by trademarks (*n* = 200) are shown in Table 4. Trademark H was only AMP-resistant, whereas A was susceptible only to AMC. Overall resistance rates were highest for the old-generation antibiotics and those for livestock use, including AMP (63%), TET (48%), STR (12.5%), and sulfonamide (13.5% and 12.5% for SX and SXT, respectively), and lowest for the antibiotics for human use, including AMC (0%), CIP (1%), and NAL (1.5%). The distribution of AMR levels between each class and the combination of antibiotic resistance in *Salmonella*-contaminated pork products is shown in Table 5. A proportion of 21 out of 200 samples (10.5%) had no resistance to any antibiotics. Most *Salmonella*-contaminated products were resistant to two classes of antibiotics (47%), followed by one class (30%), and MDR accounted for 12.5%. The combined rates of two classes of antibiotic resistance were 65.9% (83/126), 68.75% (66/96), and 54.54% (6/11) for AMP, TET, and CHL, respectively. The most common combinations of two classes of antibiotic resistance were AMP-TET (63.83%; 60/94) and AMP-(SXT-SX) (20.21%; 19/94).

Among 25 multidrug-resistant *Salmonella* isolates, 24 (96%) were resistant to the beta-lactam class (AMP), followed by tetracycline (TET), aminoglycoside (STR), and sulfonamide (SXT-SX) at 60%, 56%, and 56%, respectively. The patterns and distribution of multidrug resistance (MDR) in *Salmonella*-contaminated pork products are shown in Table 6. Eighty percent of MDR was within the three classes of antibiotic resistance. AMP-STR-TET (*n* = 5), AMP-STR-Sulfonamide (*n* = 5), and AMP-TET-Sulfonamide (*n* = 4) were the most common MDR patterns found among *Salmonella* isolates. Regarding four-class antibiotic resistance, four of five isolates (80%) were quinolone-resistant.

## 4. Discussion

The prevalence of *Salmonella* contamination in pork in this study (33.33%) was higher than that reported in many European countries (0.4–22.6%) [20], in Japan (2.5%) [21], and in the USA (8.3–10.4%) [22], but lower than that observed in Vietnam (60.5%) [11], Laos (75.27%) [23], and other regions of Thailand (41.5–64.29%) [10,23]. The differences in prevalence between Europe and the USA compared with Southeast Asia reflect variations in food safety regulations, hygiene practices, cold-chain management, and retail handling systems across regions. While this study reports *Salmonella* prevalence rather than quantitative bacterial loads (e.g., MPN/CFU), the use of qualitative data (presence/absence data) remains a standard, robust approach for establishing baseline prevalence and identifying critical contamination points within a supply chain. Notably, *Salmonella* contamination in retail stores represents an important public health concern, especially for urban consumers who predominantly purchase pork from modern retail outlets. In our study, multiple factors were associated with *Salmonella* contamination in pork. GEE analysis revealed that unpackaged pork had four times higher *Salmonella* contamination than factory-packaged pork (Table 3). The high prevalence in unpackaged pork was associated with suboptimal storage conditions and handling practices observed at the retail level. The unpackaged pork was handled directly by customers and was not consistently kept at 4 °C, thereby increasing the risk of cross- or re-contamination with *Salmonella* at the retail store [2,24]. In contrast, factory-packaged pork was prepared under GMP standards and transported to retail outlets through cold-chain logistics. In addition, factory-packaged pork was typically stored either in freezers (−18 to −20 °C) or on refrigerated shelves (2–4 °C), both of which are critical for pork quality. Refrigerated storage generally provided a maximum shelf life of approximately 8 days, whereas freezing preserved pork quality for several months [25]. However, the limitation of empirical temperature data along the cold-chain logistics process precluded a definitive conclusion regarding cold-chain integrity. The differences in storage temperature at retail stores and packaging type are associated with variations in *Salmonella* growth and contamination risk, with low storage temperatures, together with sealed packaging, associated with limiting bacterial growth and reducing cross-contamination in retail settings [2,26,27].

The marketing purpose was also significantly associated with *Salmonella* contamination in pork. The final model indicated that pork for household use had a 2.5-fold lower risk of *Salmonella* contamination than pork for restaurant use (Table 3). The demand for pork consumption among city dwellers was high, and the pork products in modern trade were varied, especially among those for household consumption. Pork marketed for household consumption generally offers a wider selection of products, including those produced under stricter hygienic standards and marketed as higher-quality or premium brands, which may contribute to lower contamination levels. In contrast, pork for restaurant use came from only two major suppliers in the study area, with most products transported from other provinces. The longer transportation distances and extended storage durations were associated with an increased risk of *Salmonella* contamination [28]. Moreover, the high local demand for pork in this area necessitated sourcing from other regions through both legal and illegal trade channels, which may further increase the risk of contamination due to variations in handling and hygiene practices [29,30].

Seasonality was another factor affecting *Salmonella* contamination in pork. In the United States, *Salmonella* prevalence in raw meat (pork, beef, chicken, turkey) is higher in the summer months than in winter [31]. Hence, the sampling period of this study was extended to encompass two full summer cycles to address the unique climatic conditions of Southern Thailand, where the summer season is relatively short (mid-March to mid-May) compared to the nine-month rainy season. This strategy was designed to ensure the proportional representation of both climatic conditions in this region. In this study, the summer season was a risk factor for increased *Salmonella* contamination in pork (Figure 3), supporting evidence from temperate regions and suggesting that warmer and drier conditions may favor bacterial persistence and transmission [32]. Moreover, previous studies have reported higher *Salmonella* rates in pig herds during the summer than in other seasons [33,34]. This may suggest that the impact of seasonal patterns on *Salmonella* contamination did not affect only pork but originated at the farm level. Additionally, the type of store was significantly associated with *Salmonella* contamination in pork. As shown in Figure 3, wholesale outlets exhibited higher levels of *Salmonella* contamination compared to minimarts and hypermarkets. This association may be driven by the fact that wholesale outlets primarily supply pork for restaurant use, which has been reported to have a four-times higher risk of contamination compared to pork intended for household consumption (Table 3). In contrast, no significant difference in *Salmonella* contamination was observed between ground and non-ground pork, suggesting that hygienic practices during the grinding process were effectively maintained.

The diversity of *Salmonella* serotypes in this study revealed that the serotype Rissen was the most frequently found in pork. In Thailand, this serotype has been reported as predominant in pig production in the northern [35] and northeastern [36] regions. Additionally, serotypes Typhimurium and Enteritidis were among the top three most common serotypes identified in our study, consistent with findings in Southeast Asia [37,38]. Although these serotypes have been reported in this region since 2000, they are also found in Europe and the United States, likely due to global pork markets [39,40]. Furthermore, these three serotypes are among the top 20 *Salmonella* serovars linked to human salmonellosis [41,42].

Antimicrobial resistance in *Salmonella* poses a serious problem in public health because this pathogen is transmitted to humans through the food chain [7]. Our findings revealed that *Salmonella* isolated from pork at retail stores exhibited a high prevalence of antimicrobial resistance. Table 4 shows the high resistance rates to AMP (beta-lactams) and TET (tetracycline) across all trademarks. Ninety-four *Salmonella* samples were resistant to two classes of antimicrobials; the most common combination was AMP-TET (Table 5). High resistance rates for AMP and TET have also been reported in the pork production chain in northeastern Thailand, Laos, and Cambodia [43,44,45]. These antimicrobials have generally been used in livestock production for disease prevention, treatment, and growth promotion over several decades, which may have contributed to the high prevalence of resistance observed [46,47].

The occurrence of MDR *Salmonella* in the livestock sector has been reported at high levels in several Southeast Asian countries, including Thailand (98%) [48], Laos (98.4%) [43], Vietnam (59.4%) [49], and Cambodia (52%) [44]. In Vietnam, MDR *Salmonella* has been detected in pork at retail stores, with prevalence ranging from 40% to 75% [49]. In contrast, our study reported a lower prevalence, with 12.5% of *Salmonella* isolated from pork in wholesale and retail stores (Table 6). Among the MDR isolates, 80% were resistance to three classes of antimicrobials, with the AMP–STR–TET resistance pattern being the most prevalent. Resistance to four antimicrobial classes was observed in 20% of MDR isolates, while resistance to five antimicrobial agents was also detected, with the predominant patterns being AMP–NOR–STR–(SX–SXT) and AMP–NOR–(SX–SXT)–TET (Table 6). These resistance patterns corresponded to antimicrobials commonly used for the treatment of diarrheal diseases in pig production in Thailand, including beta-lactams, aminoglycosides, and sulfonamides, combined with trimethoprim, quinolones, and polymyxins [50].

For a proper understanding of genetic relatedness and antibiotic resistance in *Salmonella* from pork, genomic data from Whole-Genome Sequencing (WGS) of *Salmonella* are required for further study to bridge the gap between phenotypic profiles and high-resolution genotypic characterization of this bacterial type. The genomic investigations are crucial to track the evolution and mutation of virulence factors, AMR genes, the mobilome, and resistance mechanisms, including chromosomal mutation and plasmid-mediated resistance. These high-resolution data will facilitate a comparative analysis of *Salmonella* transmission across the swine production chain and provide a deeper investigation into the spread of AMR determinants and the dynamics of cross-contamination at the retail level.

## 5. Conclusions

In conclusion, this study highlights that *Salmonella* contamination in pork from wholesale and retail outlets is influenced by multiple factors, including packaging type, marketing target, and seasonal variation. Although the overall prevalence of *Salmonella*-positive pork (33.33%) was lower than that in other regions of Thailand, unpackaged pork and pork for restaurant use were associated with an increased risk of contamination. Notably, a high AMR level was observed among the *Salmonella* isolates, while the prevalence of MDR *Salmonella* remained relatively low compared to reports from other Southeast Asian countries. These findings provide important scientific evidence for raising public health awareness regarding the safety of pork consumption among urban populations and underscore the need for improved hygiene practices and continued antimicrobial resistance surveillance.

## Figures and Tables

**Table 1:** Number of sample collections based on trademarks (A–J). HH indicates their marketing was for household use, and “No” indicates restaurant use.

Trademarks			Shop Types			Ground		Not Ground	
	HH	Total	Minimarts	Hypermarkets	Wholesale Stores	Unpack	Pack	Unpack	Pack
A	Yes	233	171	46	16	54	60	54	65
B	Yes	158	150	8	-	45	40	43	30
C	Yes	42	9	33	-	6	14	8	14
D	Yes	12	10	2	-	-	2	-	10
E	No	11	-	2	9	-	7	-	4
F	Yes	30	-	30	-	15	-	15	-
G	Yes	13	-	13	-	-	6	-	7
H	Yes	6	-	6	-	-	3	-	3
I	No	55	-	-	55	20	8	20	7
J	No	40	-	-	40	10	10	10	10

**Table 2:** Number of distributed isolates of *Salmonella* serotypes in contaminated pork products in Songkhla province based on their brand names.

Trademarks	*S*. 1,4,5,12:i:-	*S*. 4,5,12:i:-	*S*. Albany	*S*. Anatum	*S*. Brunei	*S*. Enteritidis	*S*. Krefeld	*S*. Lexington	*S*. Newport	*S*. Panama	*S.* Rissen	*S*. Stanley	*S.* Typhimurium	*S*. Weltevreden	Total
A	2	4	1	1	-	3	-	6	-	2	21	1	15	5	61
B	-	1	-	1	1	5	-	5	1	-	21	3	9	4	51
C	-	1	-	-	-	1	-	-	-	-	2	-	5	2	11
D	-	-	-	-	-	2	-	-	-	-	-	-	-	-	2
E	-	2	-	-	-	2	-	-	-	-	1	-	-	-	5
F	-	-	-	-	-	4	-	2	-	-	7	-	2	1	16
G	-	1	-	-	-	-	-	-	-	-	2	-	-	-	3
H	-	-	-	-	-	-	-	-	-	-	1	-	-	-	1
I	1	2	1	-	-	4	1	-	-	1	13	-	6	2	31
J	-	-	-	-	-	4	-	-	-	2	1	-	2	1	10
Total	3	11	2	2	1	25	1	13	1	5	78	4	39	15	200

**Table 3:** The final model for factors associated with *Salmonella* contamination in pork products in Songkhla province using repeated logistic regression (Generalized Estimating Equation, GEE).

Parameter		Estimate	Standard Error	95%	Confidence	Z	*p*-Value	Odd Ratio
				Lower	Upper			
Intercept		−0.767	0.197	−1.153	−0.380	−3.890	0.000	
Packed	No	1.460	0.169	1.129	1.791	8.640	<0.0001	4.306
	Yes	Reference						
Household	No	0.933	0.207	0.527	1.338	−4.510	<0.0001	2.542
	Yes	Reference						

**Table 4:** Percentages of antibiotic resistance in *Salmonella*-contaminated pork products collected from Songkhla province, Thailand, by brand name (*n* = 200).

Trademarks	Total (*n*)	Beta Lactam			Quinolone				Sulfonamide			
		AMC	AMP	CHL	NAL	NOR	CIP	STR	SXT	SX	TET	COL
Total (*n*)		0	126	11	3	6	2	26	27	25	96	11
A	61	0	65.57	8.20	3.28	6.56	1.64	18.03	9.84	16.39	36.07	6.56
B	51	0	56.86	3.92	1.96	1.96	0	19.61	29.41	15.69	43.14	3.92
C	11	0	45.45	9.09	0	0	0	0	0	0	36.36	18.18
D	2	0	50.00	0	0	0	0	50.00	50.00	0	100.00	0
E	5	0	80.00	0	0	0	0	0	0	0	80.00	20
F	16	0	50.00	12.50	0	6.25	6.25	0	12.5	0	43.75	0
G	3	0	66.67	0	0	0	0	0	33.33	33.33	100.00	0
H	1	0	100.00	0	0	0	0	0	0	0	100.00	0
I	31	0	70.97	0	0	0	0	9.68	0	16.13	61.29	3.23
J	19	0	73.68	5.26	0	0	0	5.26	10.53	5.26	63.16	5.26

**Table 5:** Distribution of AMR levels in each class and the combination of antibiotic resistance in *Salmonella*-contaminated pork products collected from Songkhla province, Thailand.

AMR	Total	%
All susceptible	21	10.5
Resistance to one antimicrobial class		
AMP	20	10.0
CHL	1	0.5
NAL, NOR, CIP	4	2.0
STR	5	2.5
SXT-SX	9	4.5
TET	14	7.0
COL	7	3.5
Total	60	30.0
Resistance to two antimicrobial classes		
AMP-CHL	2	1.0
AMP-STR	1	0.5
AMP-(SXT-SX)	19	9.5
AMP-TET	60	30.0
AMP-COL	1	0.5
CHL-(SXT-SX)	2	1.0
CHL-TET	2	1.0
TET-(NAL-NOR-CIP)	1	0.5
STR-(SXT-SX)	4	2.0
TET-(SXT-SX)	2	1.0
Total	94	47.0

**Table 6:** Patterns and distribution of multidrug resistance (MDR) in *Salmonella*-contaminated pork products collected from Songkhla province, Thailand.

MDR	No. of Isolates	%
Resistance to three antimicrobial classes		
AMP-CHL-SX	1	0.5
AMP-CHL-TET	1	0.5
AMP-CIP-SXT	1	0.5
AMP-COL-TET	1	0.5
AMP-NAL-STR	1	0.5
AMP-STR-(SXT-SX)	5	2.5
AMP-STR-TET	5	2.5
AMP-(SXT-SX)-TET	4	2.0
STR-SXT-TET	1	0.5
Total	20	10.0
Resistance to four antimicrobial classes		
AMP-CHL-CIP-STR	1	0.5
AMP-CHL-COL-TET	1	0.5
AMP-COL-NOR-TET	1	0.5
AMP-NOR-STR-(SX-SXT)	1	0.5
AMP-NOR-(SX-SXT)-TET	1	0.5
Total	5	2.5
Total MDR	25	12.5

## Data Availability

Most of the data in the study are contained in the article. Additional data sets are available upon reasonable request.

## Abbreviations

NTS	Nontyphoidal *Salmonella*
AMR	Antimicrobial Resistance
ISO	International Organization for Standardization
BPW	Buffered Peptone Water
MSRV	Modified Semisolid Rappaport Vassiliadis Medium
XLD	Xylose Lysine Deoxycholate
BPLS	Brilliant-Green Phenol-Red Lactose Sucrose
NA	Nutrient agar
MHA	Mueller–Hinton agar
AMP	Ampicillin
AMC	Amoxicillin/Clavulanate
CHL	Chloramphenicol
CIP	Ciprofloxacin
NAL	Nalidixic Acid
NOR	Norfloxacin
STR	Streptomycin
SX	Sulfisoxazole
TET	Tetracycline
SXT	Trimethoprim/Sulfamethoxazole
COL	Colistin
MDR	Multidrug Resistance
GMP	Good Manufacturing Practices
HH	Household

## References

[1] Lamichhane B., Mawad A.M., Saleh M., Kelley W.G., Harrington P.J., Lovestad C.W., Amezcua J., Sarhan M.M., El Zowalaty M.E., Ramadan H. (2024). Salmonellosis: An overview of epidemiology, pathogenesis, and innovative approaches to mitigate the antimicrobial resistant infections. Antibiotics.

[2] Prasertsee T., Chokesajjawatee N., Santiyanont P., Chuammitri P., Deeudom M., Tadee P., Patchanee P. (2019). Quantification and rep-PCR characterization of *Salmonella* spp. in retail meats and hospital patients in Northern Thailand. Zoonoses Public Health.

[3] Worldometer Thailand Demographics (2026): Main Cities by Population in Thailand. https://www.worldometers.info/world-population/thailand-population/.

[4] Amore G., Beloeil P.A., Boelaert F., Ferrer-Bustins N., Fierro R.G., Rizzi V., Rossi M., Stoicescu A.V., European Food Safety Authority (2026). Guidance for Reporting 2025 Data on Zoonoses, Foodborne Outbreaks and Antimicrobial Resistance. EFSA Support. Publ..

[5] Amore G., Boelaert F., Ferrer-Bustins N., Rizzi V., Rossi M., Stoicescu A.V., European Food Safety Authority (2026). Manual for Reporting on 2025 Zoonoses, Zoonotic Agents and on Some Other Pathogenic Microbiological Agents under Directive 2003/99/Ec. EFSA Support. Publ..

[6] Tast Lahti E., Karamehmedovic N., Riedel H., Blom L., Boel J., Delibato E., Denis M., van Essen-Zandbergen A., Garcia-Fernandez A., Hendriksen R. (2023). One Health surveillance—A cross-sectoral detection, characterization, and notification of foodborne pathogens. Front. Public Health.

[7] Kumar R., Adeyemi N.O., Chattaraj S., Alloun W., Thamarsha A., Anđelković S., Mitra D., Gautam P. (2025). Antimicrobial resistance in *Salmonella*: One Health perspective on global food safety challenges. Sci. One Health.

[8] Bernal J.F., Díaz P.L., Perez-Sepulveda B.M., Valencia-Guerrero M.F., Clavijo V., Wiesner M., Montaño L.A., Arevalo S.A., León I.M., Castellanos L.R. (2023). A one health approach based on genomics for enhancing the *Salmonella enterica* surveillance in Colombia. IJID Reg..

[9] Xu C., Shi Y., Zhai X., Miao X., Liu B., Kang X., Jiao X., Meng C., Pan Z. (2025). *Salmonella* Prevalence in Carcasses and Cross-Contamination Risk Factors in Pig Slaughterhouses: A Systematic Evaluation and Meta-Analysis. Foodborne Pathog. Dis..

[10] Patchanee P., Tansiricharoenkul K., Buawiratlert T., Wiratsudakul A., Angchokchatchawal K., Yamsakul P., Yano T., Boonkhot P., Rojanasatien S., Tadee P. (2016). *Salmonella* in pork retail outlets and dissemination of its pulsotypes through pig production chain in Chiang Mai and surrounding areas, Thailand. Prev. Vet. Med..

[11] Ngo H.H.T., Nguyen-Thanh L., Pham-Duc P., Dang-Xuan S., Le-Thi H., Denis-Robichaud J., Nguyen-Viet H., Le T.T., Grace D., Unger F. (2021). Microbial contamination and associated risk factors in retailed pork from key value chains in Northern Vietnam. Int. J. Food Microbiol..

[12] Prasertsee T., Prachantasena S., Tantitaveewattana P., Chuaythammakit P., Pascoe B., Patchanee P. (2024). Assessing antimicrobial resistance profiles of *Salmonella enterica* in the pork production system. J. Med. Microbiol..

[13] Dang-Xuan S., Nguyen-Viet H., Pham-Duc P., Unger F., Tran-Thi N., Grace D., Makita K. (2019). Risk factors associated with *Salmonella* spp. prevalence along smallholder pig value chains in Vietnam. Int. J. Food Microbiol..

[14] Yokozawa T., Dang-Xuan S., Nguyen-Viet H., Lapar L., Makita K. (2016). Transition of *Salmonella* prevalence in pork value chain from pig slaughterhouses to markets in Hung Yen, Vietnam. J. Vet. Epidemiol..

[15] Nhung N.T., Van N.T.B., Van Cuong N., Duong T.T.Q., Nhat T.T., Hang T.T.T., Nhi N.T.H., Kiet B.T., Hien V.B., Ngoc P.T. (2018). Antimicrobial residues and resistance against critically important antimicrobials in non-typhoidal *Salmonella* from meat sold at wet markets and supermarkets in Vietnam. Int. J. Food Microbiol..

[16] Phan T.T., Khai L.T.L., Ogasawara N., Tam N.T., Okatani A.T., Akiba M., Hayashidani H. (2005). Contamination of *Salmonella* in retail meats and shrimps in the Mekong Delta, Vietnam. J. Food Prot..

[17] (2017). Microbiology of the Food Chain—Horizontal Method for the Detection, Enumeration and Serotyping of Salmonella—Part 1: Detection of *Salmonella* spp..

[18] CLSI (2018). Performance Standards for Antimicrobial Susceptibility Testing.

[19] EUCAST Clinical Breakpoints and Epidemiological Cut-Off Values for the Priority List of Antimicrobials to Be Tested for *Salmonella enterica*. https://www.ecdc.europa.eu/sites/default/files/documents/antimicrobial-resistance-Salmonella-Campylobacter-harmonised-monitoring-Annex-Aug-2021.pdf.

[20] Roasto M., Bonardi S., Mäesaar M., Alban L., Gomes-Neves E., Vieira-Pinto M., Vågsholm I., Elias T., Lindegaard L.L., Blagojevic B. (2023). *Salmonella enterica* prevalence, serotype diversity, antimicrobial resistance and control in the European pork production chain. Trends Food Sci. Technol..

[21] Sasaki Y., Ohya K., Momose Y., Uema M., Ikeda T., Sasaki M., Asai T. (2025). Serovars and Antimicrobial Resistance of *Salmonella* in Food Workers and Livestock Products: Insights into Foodborne Transmission Pathways in Eastern Japan. Pathogens.

[22] Duffy E., Belk K., Sofos J., Bellinger G., Pape A., Smith G. (2001). Extent of microbial contamination in United States pork retail products. J. Food Prot..

[23] Meunsene D., Eiamsam-Ang T., Patchanee P., Pascoe B., Tadee P., Tadee P. (2021). Molecular evidence for cross boundary spread of *Salmonella* spp. in meat sold at retail markets in the middle Mekong basin area. PeerJ.

[24] Dang-Xuan S., Nguyen-Viet H., Pham-Duc P., Grace D., Unger F., Nguyen-Hai N., Nguyen-Tien T., Makita K. (2018). Simulating cross-contamination of cooked pork with *Salmonella enterica* from raw pork through home kitchen preparation in Vietnam. Int. J. Environ. Res. Public Health.

[25] Custódio F.B., Vasconcelos-Neto M.C., Theodoro K.H., Chisté R.C., Gloria M.B.A. (2018). Assessment of the quality of refrigerated and frozen pork by multivariate exploratory techniques. Meat Sci..

[26] Mann J., Smith L., Brashears M. (2004). Validation of time and temperature values as critical limits for *Salmonella* and background flora growth during the production of fresh ground and boneless pork products. J. Food Prot..

[27] Silva J.L.d., Vieira B.S., Carvalho F.T., Carvalho R.C.T., Figueiredo E.E.d.S. (2022). *Salmonella* behavior in meat during cool storage: A systematic review and meta-analysis. Animals.

[28] Zhong J., Zhou G., Yang Y., Sun X., Zhang H., Qu X., Su Q., Chen Q., Niu B. (2024). Quantitative risk assessments of *Salmonella* spp. in domestic pork in China. Braz. J. Microbiol..

[29] Fanelli A., Muñoz O., Mantegazza L., De Nardi M., Capua I. (2022). Is the COVID-19 pandemic impacting on the risk of African Swine Fever virus (ASFV) introduction into the United States? A short-term assessment of the risk factors. Transbound. Emerg. Dis..

[30] Soon J.M., Manning L. (2018). Food smuggling and trafficking: The key factors of influence. Trends Food Sci. Technol..

[31] Williams M.S., Ebel E.D., Golden N.J., Schlosser W.D. (2014). Temporal patterns in the occurrence of *Salmonella* in raw meat and poultry products and their relationship to human illnesses in the United States. Food Control.

[32] Balta I., Lemon J., Murnane C., Pet I., Vintila T., McCleery D., Callaway T., Douglas A., Stef L., Corcionivoschi N. (2024). The One Health aspect of climate events with impact on foodborne pathogens transmission. One Health.

[33] Farzan A., Friendship R., Cook A., Pollari F. (2010). Occurrence of *Salmonella*, *Campylobacter*, *Yersinia enterocolitica*, *Escherichia coli* O157 and *Listeria monocytogenes* in swine. Zoonoses Public Health.

[34] Ainslie-Garcia M.H., Farzan A., Newman J.E., Friendship R.M., Lillie B.N. (2018). *Salmonella* fecal shedding in pigs from birth to market and its association with the presence of *Salmonella* in palatine tonsils and submandibular lymph nodes at slaughter. Can. J. Vet. Res..

[35] Tadee P., Kumpapong K., Sinthuya D., Yamsakul P., Chokesajjawatee N., Nuanualsuwan S., Pornsukarom S., Molla B.Z., Gebreyes W.A., Patchanee P. (2014). Distribution, quantitative load and characterization of *Salmonella* associated with swine farms in upper-northern Thailand. J. Vet. Sci..

[36] Phongaran D., Khang-Air S., Angkititrakul S. (2019). Molecular epidemiology and antimicrobial resistance of *Salmonella* isolates from broilers and pigs in Thailand. Vet. World.

[37] Bescucci D.M., Moote P.E., Ortega Polo R., Uwiera R.R., Inglis G.D. (2020). *Salmonella enterica* serovar Typhimurium temporally modulates the enteric microbiota and host responses to overcome colonization resistance in swine. Appl. Environ. Microbiol..

[38] Patra S.D., Mohakud N.K., Panda R.K., Sahu B.R., Suar M. (2021). Prevalence and multidrug resistance in *Salmonella enterica* Typhimurium: An overview in South East Asia. World J. Microbiol. Biotechnol..

[39] Broadway P.R., Brooks J.C., Mollenkopf D.F., Calle M.A., Loneragan G.H., Miller M.F., Carroll J.A., Sanchez N.C.B., Wittum T.E. (2021). Prevalence and antimicrobial susceptibility of *Salmonella* serovars isolated from US retail ground pork. Foodborne Pathog. Dis..

[40] D’Incau M., Salogni C., Giovannini S., Ruggeri J., Scali F., Tonni M., Formenti N., Guarneri F., Pasquali P., Alborali G.L. (2021). Occurrence of *Salmonella* Typhimurium and its monophasic variant (4,[5], 12: i:-) in healthy and clinically ill pigs in northern Italy. Porc. Health Manag..

[41] EFSA (2021). The European Union one health 2019 zoonoses report. EFSA J..

[42] Xu X., Biswas S., Gu G., Elbediwi M., Li Y., Yue M. (2020). Characterization of multidrug resistance patterns of emerging *Salmonella enterica* serovar Rissen along the food chain in China. Antibiotics.

[43] Sinwat N., Angkittitrakul S., Coulson K.F., Pilapil F.M.I.R., Meunsene D., Chuanchuen R. (2016). High prevalence and molecular characteristics of multidrug-resistant *Salmonella* in pigs, pork and humans in Thailand and Laos provinces. J. Med. Microbiol..

[44] Trongjit S., Angkititrakul S., Tuttle R.E., Poungseree J., Padungtod P., Chuanchuen R. (2017). Prevalence and antimicrobial resistance in *Salmonella enterica* isolated from broiler chickens, pigs and meat products in Thailand–Cambodia border provinces. Microbiol. Immunol..

[45] Chea B., Kong S., Thim S., Ban N., Chrun R., Venn V., Fernandez-Colorado C., Kang K. (2025). Prevalence and antimicrobial resistance of *Salmonella* spp. isolated from swine and poultry farms in Cambodia. Vet. World.

[46] Enshaie E., Nigam S., Patel S., Rai V. (2025). Livestock Antibiotics Use and Antimicrobial Resistance. Antibiotics.

[47] Kuswandi B., Futra D., Heng L., Oprea E.A., Grumezescu M.A. (2017). Chapter 15—Nanosensors for the Detection of Food Contaminants. Nanotechnology Applications in Food.

[48] Tu L., Hoang N., Cuong N., Campbell J., Bryant J., Hoa N., Kiet B., Thompson C., Duy D., Phat V. (2015). High levels of contamination and antimicrobial-resistant non-typhoidal *Salmonella* serovars on pig and poultry farms in the Mekong Delta of Vietnam. Epidemiol. Infect..

[49] Holohan N., Wallat M., Hai Yen Luu T., Clark E., Truong D.T.Q., Xuan S.D., Vu H.T.K., Van Truong D., Tran Huy H., Nguyen-Viet H. (2022). Analysis of antimicrobial resistance in non-typhoidal *Salmonella* collected from pork retail outlets and slaughterhouses in Vietnam using whole genome sequencing. Front. Vet. Sci..

[50] Nguyet L.T.Y., Keeratikunakorn K., Kaeoket K., Ngamwongsatit N. (2022). Antibiotic resistant *Escherichia coli* from diarrheic piglets from pig farms in Thailand that harbor colistin-resistant mcr genes. Sci. Rep..

